# QuEChERS-超高效液相色谱-串联质谱法测定果蔬中79种农药残留

**DOI:** 10.3724/SP.J.1123.2025.02015

**Published:** 2025-09-08

**Authors:** Le ZHAO, Xianjun LIU, Hao ZHANG, Jian LI, Liang CAI, Xiang FAN, Tanyao LI, Dongyang CHEN

**Affiliations:** 1.南华大学公共卫生学院，湖南 衡阳 421001; 1. School of Public Health，University of South China，Hengyang 421001，China; 2.湖南省疾病预防控制中心（湖南省预防医学科学院），湖南 长沙 410153; 2. Hunan Provincial Center for Disease Control and Prevention （Hunan Academy of Preventive Medicine），Changsha 410153，China

**Keywords:** 农药残留, 超高效液相色谱-串联质谱法, QuEChERS, 风险评估, pesticide residues, ultra performance liquid chromatography-tandem mass spectrometry （UPLC-MS/MS）, QuEChERS, risk assessment

## Abstract

果蔬中农药残留是影响农产品质量和消费者身体健康的热点问题，如何有效监测和控制农药残留已成为食品安全领域的重要课题。通过优化QuEChERS前处理方法及仪器测定参数，建立了果蔬中有机磷类、氨基甲酸酯类、拟除虫菊酯类等79种典型农药的QuEChERS-超高效液相色谱-串联质谱（QuEChERS-UPLC-MS/MS）同时测定方法。果蔬样品经均质后在4 ℃条件下用乙腈提取，QuEChERS法净化并离心过滤后进行UPLC-MS/MS分析。采用ACQUITY UPLC HSS T3（100 mm×2.1 mm，1.8 μm）色谱柱分离，以水-甲醇（98∶2，v/v，含5 mmol/L乙酸铵和0.1%甲酸）为水相，甲醇-水（98∶2，v/v，含5 mmol/L乙酸铵和0.1%甲酸）为有机相进行梯度洗脱。采用三重四极杆质谱仪，在电喷雾离子源正离子扫描模式下，基质匹配标准曲线法定量。结果表明，在优化的条件下，目标化合物在0.1**~**200 μg/L范围内线性关系良好，相关系数（*r*）均大于0.990；79种化合物的检出限（LOD）和定量限（LOQ）范围分别为0.01~4.0 μg/kg和0.03~13.0 μg/kg，3个添加水平下回收率为 78.2%~119.8%，相对标准偏差（RSD）均小于15.8%。采用建立的方法对80份果蔬样本进行检测，31份样本中检出19种农药，以噻虫胺、啶虫脒和噻虫嗪检出率最高，含量为0.012~2.62 mg/kg；对检出的烟碱类农药（噻虫胺和噻虫嗪）进行了初步风险评估，结果显示，噻虫胺在水果和蔬菜中的慢性膳食摄入风险值（%ADI）分别为5.74%和0.36%，噻虫嗪在水果和蔬菜中的%ADI分别为0.40%、19.50%，二者在蔬菜和水果中的%ADI均低于100%，均在可接受范围内。该方法简便、灵敏、准确，适用于果蔬中多种农药残留的同时测定。

## Abstract (en)

Pesticide residues in fruits and vegetables are becoming a serious issue. These residues can affect the quality of agricultural products and people’s health. Therefore， it has become crucial to effectively monitor and control pesticide residues in the food safety field. In this study， a rapid and effective QuEChERS-ultra performance liquid chromatography-tandem mass spectrometry （QuEChERS-UPLC-MS/MS） method was established for the simultaneous determination of 79 typical pesticides in vegetables and fruits， including organophosphates， carbamates， and pyrethroids. The pretreatment， UPLC， and MS/MS conditions were optimized. The fruit and vegetable samples were extracted with frozen acetonitrile after pulverization and homogenization， cleaned up by the QuEChERS method， filtered through a centrifugal membrane， and analyzed by UPLC-MS/MS. The separation was carried out on an ACQUITY UPLC HSS T3 column （100 mm×2.1 mm， 1.8 μm） with gradient elution. The aqueous and organic phases were water-methanol （98∶2， v/v） and methanol-water （98∶2， v/v） respectively， both with 5 mmol/L ammonium acetate and 0.1% formic acid. A triple quadrupole mass spectrometer was used in positive-ion electrospray ionization （ESI^+^） scanning mode， with target pesticide residues quantified using the matrix-matched standard-curve method. The results showed that under the optimized conditions， the 79 target compounds were determined with good linearities in the range of 0.1-200 μg/L， and the correlation coefficients （*r*） were all greater than 0.990. The limits of detection （LODs） and limits of quantification （LOQs） of the 79 compounds were in the range of 0.01-4.0 μg/kg and 0.03-13.0 μg/kg. The recoveries at three spiked levels ranged from 78.2% to 119.8%， with relative standard deviations （RSDs） all less than 15.8%. The established method was successfully applied to 80 samples of fruits and vegetables from Hunan province. As a result， 19 pesticides were detected in 31 samples， and thiamethoxam， acetamiprid and clothianidin being the most highly detected with a content range of 0.012-2.62 mg/kg； According to the data of the Hunan province survey yearbook， the percentages of acceptable daily intake （%ADI） for chronic dietary exposure of the detected neonicotinoid insecticides （thiamethoxam and clothianidin） have been calculated. The results indicate that the %ADI of clothianidin in fruits and vegetables ranged from 5.74% to 0.36%， respectively， and the %ADI of thiamethoxam in fruits and vegetables ranged from 0.40% to 19.50%. The %ADI of both pesticides were found to be less than 100%， indicating they are within acceptable limits. The method is simple， sensitive， accurate， and suitable for the simultaneous determination of multiple pesticide residues in fruits and vegetables.

农产品中农药残留一直广受公共卫生领域及公众的关注，国家统计数据显示，我国农药年均使用量50万吨以上，大部分用于果蔬种植领域，由农药超量、滥用造成果蔬农药残留超标的问题已成为食品行业持续关注的热点^［1］^。果蔬中农药残留不仅会降低果蔬的质量，更对人体健康产生急慢性危害，农药成分及其代谢物还可以通过富集和生物积累的方式在食物链内迁移，是目前广受关注的新污染物^［2］^。世界各国对农产品中农药残留要求严格，日本在2006年实施的“肯定列表制度”就已经涉及了51 392项农药残留标准，2023年欧盟（EC） No 396/2005设置了约15万项农药残留标准，而目前我国现行的GB 2763-2021涉及的农药残留标准仅10 092项，导致我国农产品常因农药残留问题遭到出口阻碍，造成经济损失^［3］^。因此，发展快速高效、灵敏准确的多类别多组分农药残留测定方法对保障食品安全极其必要。

当前农药残留的检测方法主要有液相色谱法（LC）^［4］^、气相色谱-质谱法（GC-MS）^［5］^和液相色谱-串联质谱法（LC-MS/MS）^［6］^等。Lima等^［7］^利用GC-MS测定了葡萄样品中的有机磷和有机氯农药，方法检出限为0.002 5~0.02 mg/kg，回收率为70%~125%；Almeida 等^［8］^使用LC-MS/MS检测了蜂蜜中127种多类别农药残留，样品检出限低至0.1 μg/kg。此外，在农药残留测定中样品前处理通常需包含净化步骤以去除基质干扰，常见的净化技术有固相萃取^［9］^、液液萃取^［10］^和QuEChERS法^［11，12］^等。其中QuEChERS法结合了液液萃取和基质分散固相萃取技术，减少了溶剂用量，具有快速、简便、廉价等优点^［13］^，已广泛应用于包括农药残留监测的各个领域。但当前国内外针对农药残留的研究主要集中在某一类高风险、新型农药上^［14，15］^，对具有地域性特点的多种类多组分农药关注不足，本研究以目前生产量大、使用量大、检出率高的79种农药（包括有机磷类、氨基甲酸酯类杀虫剂、杀菌剂、除草剂、新型烟碱类和多种我国尚未确定最高残留量的农药）为研究对象，以QuEChERS前处理结合超高效液相色谱-串联质谱法（UPLC-MS/MS），通过优化前处理程序和仪器条件，系统考察方法性能，建立了果蔬中79种农药残留的快速检测方法。同时采集某地区果蔬进行测定，并进行初步风险评估，以此了解果蔬潜在的农药残留安全问题，提升质量安全监管的针对性与有效性，能为果蔬生产种植过程中质量安全标准及风险管理措施的制定提供参考依据。

## 1 实验部分

### 1.1 仪器、试剂与材料

Xevo TQ-S超高效液相色谱-串联质谱仪（美国Waters公司）；MS204S电子天平（精度0.000 01 g，瑞士梅特勒-托利多公司）；XW-80A涡旋混匀器（中国其林贝尔仪器制造有限公司）；TG20高速冷冻离心机（长沙英泰仪器有限公司）；Elma-S300H超声波清洗仪（德国Elma公司）；Milli-Q超纯水仪（美国Millipore公司）。

乙腈、甲醇、乙酸铵（色谱级，德国Merck公司）；甲酸（色谱纯，德国Fluka公司）；硫酸镁、氯化钠、柠檬酸钠、柠檬酸氢二钠（分析纯，国药集团化学试剂有限公司）；*N*-丙基乙二胺（PSA）（40~60 μm，德国Merck公司）；有机微孔滤膜（0.22 μm，上海安谱实验科技股份有限公司）；Solvs-UP3溶剂效应消除器（纳鸥科技有限公司）。

76种农药混合标准溶液（批号：IST024462-10A，质量浓度：10 μg/mL，溶剂为乙腈）、联苯肼酯乙腈溶液（批号：1ST25030-100A，质量浓度：100 μg/mL）、丁氟螨酯（批号：1ST25064，纯度：98%）、四满嗪丙酮溶液（批号：1ST25055-100B，质量浓度：100 μg/mL），均采购于天津阿尔塔科技有限公司；实验用水为超纯水；果蔬样品购于当地超市和零售店。

### 1.2 样品制备

称取10 g均质后果蔬样品于50 mL塑料离心管中，加入10 mL 4 ℃乙腈振荡1 min，然后分别加入4 g无水硫酸镁、1 g氯化钠、1 g柠檬酸钠、0.5 g柠檬酸氢二钠及1颗陶瓷均质子，剧烈振荡1 min后，4 500 r/min离心5 min。吸取2 mL上清液至内含除水剂和净化材料的塑料离心管中（每毫升提取液使用150 mg无水硫酸镁，25 mg PSA），涡旋混匀1 min，4 500 r/min离心5 min，吸取上清液1 mL过0.22 μm微孔滤膜，上机测定。

### 1.3 标准溶液的配制

称取丁氟螨酯10 mg（精确至0.000 01 g）至10 mL容量瓶中，以乙腈溶解并定容至刻度，摇匀，制得1 000 μg/mL的丁氟螨酯标准溶液，-20 ℃保存备用。

取76种农药混合标准品1 mL，联苯肼酯标准溶液、四满嗪标准溶液各0.1 mL，丁氟螨酯标准溶液10 μL，置于10 mL容量瓶中，用乙腈稀释并定容至刻度，摇匀，制成1 μg/mL混合标准储备溶液，-20 ℃保存备用。

取空白样品红提，按照1.2节方法处理，得到基质提取溶液。以基质提取溶液为稀释液对混合标准储备液进行逐级稀释，配制成系列浓度的基质匹配标准曲线。

### 1.4 测定条件

#### 1.4.1 色谱条件

ACQUITY UPLC HSS T3色谱柱（100 mm×2.1 mm，1.8 μm）；流动相：A相为水-甲醇（98∶2，v/v，含5 mmol/L乙酸铵和0.1%甲酸），B相为甲醇-水（98∶2，v/v，含5 mmol/L乙酸铵和0.1%甲酸）；流速：0.3 mL/min；柱温：35 ℃；进样量：2 μL。流动相洗脱程序：0~2.7 min，100%A~80%A；2.7~3.7 min，80%A~60%A；3.7~7 min，60%A~0%A；7~8.6 min，0%A；8.6~9 min，0A~100%A；9~10 min，100%A。

#### 1.4.2 质谱条件

质谱条件：电喷雾离子（ESI）源，正、负离子同时扫描；多反应监测模式（MRM）；毛细管电压：3.00 kV（ESI^+^）、2.50 kV（ESI^-^）；离子源温度150 ℃；锥孔气流量150 L/h；脱溶剂气温度500 ℃；脱溶剂气流量1000 L/h。其他质谱条件见表1。

**表1:** 79种农药的质谱信息

Compound	Parent ion （*m/z*）	Daughter ions （*m/z*）	Collision energies/eV	Cone voltage/V
Methamidophos （甲胺磷）	142.0	94.0^*^， 125.0	15， 15	25
Methomyl （灭多威）	163.0	88.1^*^， 106.1	10， 10	30
Metolcarb （速灭威）	166.0	109.1^*^， 94.1	12， 27	30
Cyromazine （灭蝇胺）	167.0	85.0^*^， 108.0	20， 21	30
XMC emulsion （灭除威）	180.1	123.0^*^， 95.0	10， 21	25
Acephate （乙酰甲胺磷）	184.0	143.0^*^， 125.0	8， 20	18
Carbendazim （多菌灵）	192.0	160.0^*^， 132.0	18， 30	25
Diethyltoluamide （避蚊胺）	192.3	91.5^*^， 65.1	31， 47	30
Isoprocarb （异丙威）	194.1	95.0^*^， 137.1	14， 10	25
Pyrimethanil （嘧霉胺）	200.0	106.9^*^， 168.1	25， 35	35
Carbaryl （甲萘威）	202.1	145.1^*^， 127.0	29， 22	25
Dinotefuran （呋虫胺）	203.0	157.0^*^， 129.0	10， 20	15
Aldicarb sulfoxid （涕灭威亚枫）	207.0	89.1^*^， 132.1	15， 10	20
Propoxur （残杀威）	210.1	111.1^*^， 168.0	15， 10	30
Aldicarb （涕灭威）	213.0	89.1^*^， 98.0	10， 5	15
Omethoate （氧乐果）	214.0	182.9^*^， 124.9	11， 22	30
Atrazine （莠去津）	216.0	174.0^*^， 132.0	18， 15	25
Carbofuran （克百威）	222.1	123.1^*^， 165.1	17， 5	30
Aldicarb-sulfone （涕灭威砜）	223.0	86.0^*^， 76.0	20， 20	30
Acetamiprid （啶虫脒）	223.1	126.0^*^， 56.2	17， 13	30
Monocrotophos （久效磷）	224.1	127.0^*^， 98.1	16， 15	26
Mevinphos 1 （速灭磷1）	225.0	127.0^*^， 193.0	10， 15	20
Mevinphos 2 （速灭磷2）	225.0	127.0^*^， 193.0	10， 15	25
Mercaptodimethur （甲硫威）	226.0	169.1^*^， 121.1	10， 22	30
Picaridin （埃卡瑞丁）	230.0	130.0^*^， 95.0	15， 35	30
Dimethoate （乐果）	230.0	199.0^*^， 125.0	10， 10	24
3-Hydroxycarbofuran （3-羟基克百威）	238.0	181.0^*^， 163.0	12， 20	34
Pirimicarb （抗蚜威）	239.1	72.1^*^， 182.1	35， 20	30
Clomazone （异噁草酮）	240.0	125.1^*^， 89.1	17， 46	30
Clothianidin （噻虫胺）	250.0	169.0^*^， 132.0	14， 18	30
Thiacloprid （噻虫啉）	253.0	126.0^*^， 90.1	17， 45	30
Metasystox （甲基内吸磷）	253.0	89.0^*^， 61.0	10， 35	30
Imidacloprid （吡虫啉）	256.0	175.0^*^， 209.0	20， 16	30
Phorate （甲拌磷）	261.1	75.1^*^， 199.0	15， 5	15
Imidaclothiz （氯噻啉）	262.0	181.0^*^， 122.0	15， 30	30
Demeton-*S*-methyl sulfone （内吸磷-*S*-甲基砜）	263.0	109.0^*^， 169.0	25， 9	30
Nitenpyram （烯啶虫胺）	271.0	126.0^*^， 99.0	20， 22	30
Cadusafos （硫线磷）	271.0	159.0^*^， 131.0	15， 20	20
Phorate sulfoxide （甲拌磷亚砜）	277.0	143.0^*^， 171.0	20， 18	30
Sulfoxaflor （氟啶虫胺腈）	278.0	173.9^*^， 153.9	10， 30	25
Metalaxyl （甲霜灵）	280.3	220.2^*^， 192.1	15， 20	30
Dodemorph （吗菌灵）	282.0	116.0^*^， 98.0	20， 30	30
Pendimethalin （二甲戊灵）	282.0	212.0^*^， 194.0	5， 15	25
Vamidothion （蚜灭磷）	288.0	146.0^*^， 118.0	15， 18	30
Flupyradifurone （氟吡呋喃酮）	289.1	126.1^*^， 90.0	20， 40	30
Myclobutanil （腈菌唑）	289.1	70.1^*^， 125.1	17， 33	30
Thiamethoxam （噻虫嗪）	292.0	211.0^*^， 132.0	18， 21	30
Phorate sulfone （甲拌磷砜）	293.0	171.0^*^， 143.0	15， 15	25
Triadimefon （三唑酮）	294.2	197.0^*^， 225.2	15， 13	30
Mefenacet （苯噻酰草胺）	299.0	148.1^*^， 120.1	18， 25	40
Bifenazate （联苯肼酯）	301.0	170.0^*^， 198.0	20， 5	25
Clofentezine （四满嗪）	303.0	138.1^*^， 102.1	9， 20	25
Fenamiphos （苯线磷）	304.1	271.1^*^， 202.1	21， 37	30
Tebuconazole （戊唑醇）	308.2	70.1^*^， 125.0	21， 37	30
Azinphos-methyl （甲基谷硫磷）	318.0	132.0^*^， 160.0	5， 10	15
Fenamiphos sulfoxide （苯线磷亚砜）	320.1	233.0^*^， 108.1	20， 45	15
Sulfadiazine （磺胺嘧啶）	323.2	126.0^*^， 277.0	40， 10	30
Benalaxyl （苯霜灵）	326.0	148.0^*^， 294.0	10， 5	40
Pyrethrin （除虫菊素）	329.2	161.1^*^， 133.1	10， 15	25
Pirimiphos ethyl （嘧啶磷）	334.0	198.0^*^， 182.0	20， 25	40
Fenamiphos sulfone （苯线磷砜）	336.0	266.0^*^， 188.0	20， 25	15
Propiconazole （丙环唑）	342.0	159.0^*^， 69.0	20， 20	30
Thiophanate-methyl （甲基硫菌灵）	343.0	151.0^*^， 93.0	20， 45	25
Oxazone （噁草酮）	345.1	220.0^*^， 303.0	17， 5	40
Etoxazole （乙螨唑）	360.0	141.1^*^， 304.3	33， 17	40
Sanmite （哒螨灵）	365.0	147.0^*^， 309.0	20， 10	30
Paichongding 1 （派虫啶1）	367.2	263.1^*^， 137.1	15， 25	30
Paichongding 2 （派虫啶2）	367.2	263.1^*^， 137.1	15， 25	30
Propargite （炔螨特）	368.0	175.1^*^， 231.1	9， 1	30
Prochloraz （嘧酰胺）	376.0	308.0^*^， 266.0	10， 15	25
Pyraclostrobin （吡唑醚菌酯）	388.0	163.1^*^， 194.1	17， 5	30
Dimethomorph 2 （烯酰吗啉2）	388.1	301.1^*^， 165.1	17， 33	30
Dimethomorph 1 （烯酰吗啉1）	388.3	301.1^*^， 165.0	20， 35	30
Difenoconazole （苯醚甲环唑）	406.0	251.1^*^， 111.1	25， 69	30
Spirodiclofen （螺螨酯）	411.0	313.0^*^， 71.1	10， 15	30
Cyflumetofen （丁氟螨酯）	448.0	173.1^*^， 249.2	25， 2	30
Abamectin （阿维菌素）	895.5	751.3^*^， 327.2	45， 50	90
Fluazinam （氟定胺）	462.9	415.9^*^， 397.9	-13， -13	30
Demeton （内吸磷）	259.0	89.0^*^， 61.0	10， 35	30

* Quantitative ion. The numbers 1 and 2 indicate isotopes.

## 2 结果和讨论

### 2.1 实验条件考察

#### 2.1.1 样品前处理条件优化

为提高萃取效率，减少基质干扰，萃取溶剂应与农药极性相近。与其他溶剂相比，乙腈极性（中高极性）与大多数农药化合物极性相近，受脂质和蛋白质的干扰更少，共萃取的基质成分也更少，故选择乙腈作为提取溶剂。水果和蔬菜样品中存在大量水分、色素以及糖类，加入4 g无水硫酸镁吸水可减少乙腈相中的水分，同时为避免无水硫酸镁吸水放热带来的影响，使用冷藏乙腈比常温乙腈效率更高；加入1 g氯化钠通过盐析效应促使农药进入有机层。另外，在提取过程中加入柠檬酸盐作为缓冲盐有利于提取pH敏感性农药^［16］^。

将提取液进一步采用无水硫酸镁除水，可使提取液极性降低，促使某些极性基质共萃取物沉淀；PSA是一种弱阴离子交换剂，具有极性作用和弱阴离子交换作用，可以去除各种极性有机酸、极性色素、糖、脂肪酸和其他一些形成氢键的基质共提取物^［17］^，但使用量过大会吸附强极性目标物。进一步考察了PSA的使用量（每毫升提取液分别使用10、15、25、30 mg PSA）对待测物回收率的影响，结果显示4种PSA使用量的回收率分别为56%~121%、64%~128%、79%~116%、74%~119%，因此采用每毫升提取液使用25 mg PSA。此外，石墨化炭黑（GCB）常在QuEChERS法中使用以去除色素，但GCB易吸附平面环状结构的农药如嘧霉胺等从而导致目标物损失^［18］^，试验比较了添加GCB对待测物回收率的影响，发现在使用GCB时嘧霉胺、联苯肼酯、阿维菌素的回收率分别降低至25.1%、40.5%和58.7%，因此本研究不添加GCB。

#### 2.1.2 仪器条件优化

溶剂效应易造成色谱峰展宽，保留时间漂移^［19］^，由于样品中的农药残留含量低，轻微波动可对样品分子在色谱柱内的浓度分布造成干扰，对待测物的定量造成极大影响。实验过程中发现甲胺磷极性较强，在色谱柱上的保留差，受溶剂效应干扰大，导致峰分叉；乙酰甲胺磷、氧乐果等组分分离度较差；通过使用溶剂效应消除器，较好地解决了甲胺磷的峰形问题以及其他物质的分离问题，见图1。

**图1:**
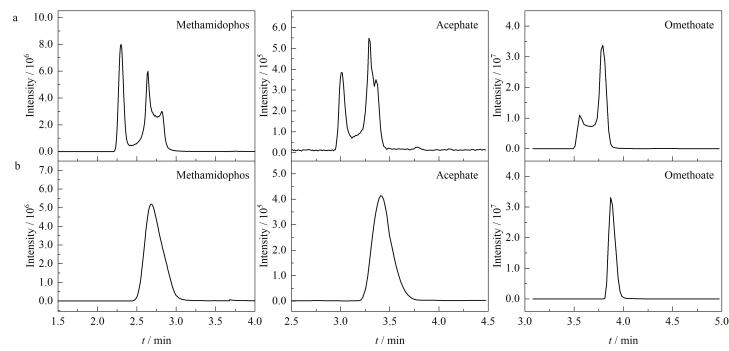
甲胺磷、乙酰甲胺磷和氧乐果在溶剂消除器（a）使用前和（b）使用后的总离子流图

比较了BEH C18（100 mm×2.1 mm，1.7 μm）和HSS T3（100 mm×2.1 mm，1.8 μm）两种色谱柱对待测物的分离效果，结果表明，二者虽均为反相色谱柱，但HSS T3色谱柱通过高密度键合C18和彻底封尾技术使其对极性农药（如甲胺磷、乙酰甲胺磷）的保留更强，同时，其特殊的表面化学修饰使其在高水含量流动相中更稳定，能有效减少强极性农药的峰形问题。在极性范围较广的79种化合物分析中，其保留性能、峰形表现以及化合物响应均优于常规C18色谱柱，因此选择HSS T3柱作为分析柱。79种农药混合标准溶液的总离子流图（TIC）见图2。

**图2:**
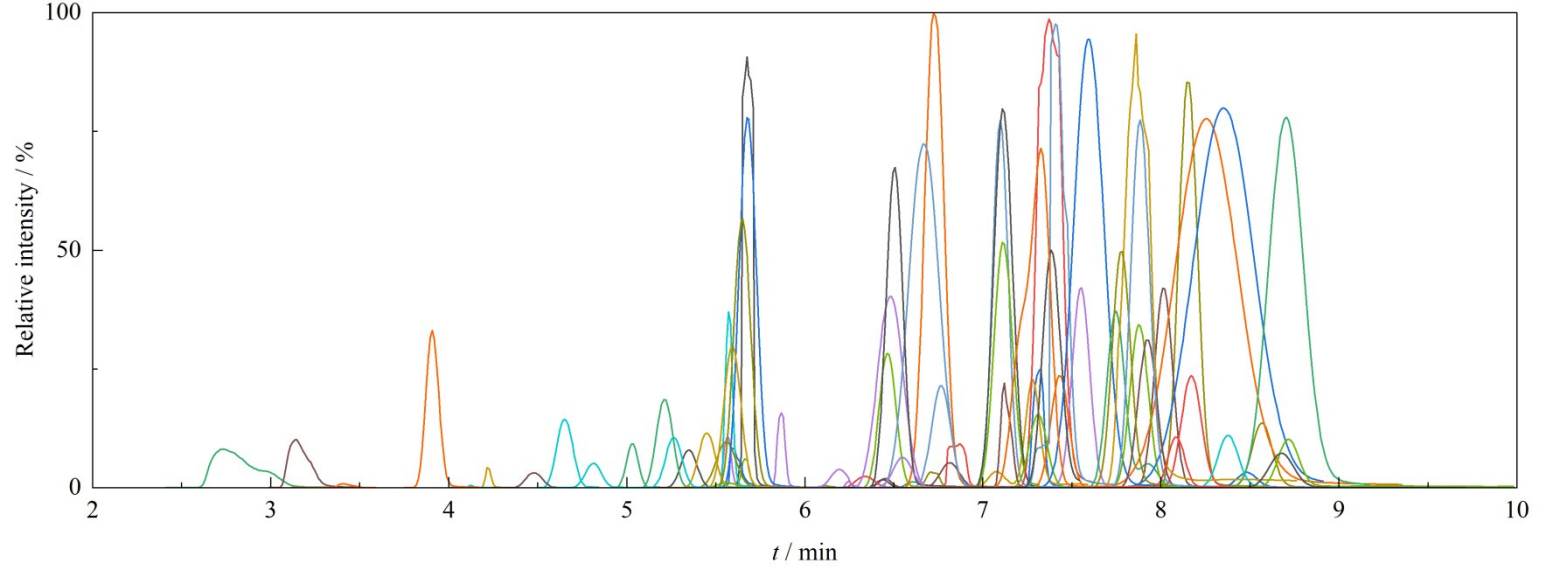
79种农药混合标准溶液（50 μg/L）总离子流图

### 2.2 方法学考察

#### 2.2.1 **基质效应**（ME）

以番茄、红提为典型样品，通过比较空白基质与空白溶剂中待测物的响应值来评价基质效应，计算公式为ME=*A/B*×100%，式中*A*为基质样品添加待测物中的响应值，*B*为空白溶剂中添加相同含量待测物的响应值，当ME<100%时，为基质抑制效应，当ME>100%时，为基质增强效应；ME>150%或<50%时表示强基质效应，120%<ME≤150%或50%≤ME<80%表示中等基质效应，80%≤ME≤120%时为弱基质效应^［20］^。79种农药的基质效应见附表1（www.chrom-China.com）。由结果可知，番茄基质中呋虫胺、氧乐果、烯啶虫胺、吗菌灵等物质为强基质效应，其余大部分目标物表现为中等基质效应；红提基质中大部分物质为中等基质效应。因此，采用基质匹配标准曲线进行定量，以消除基质效应。

#### 2.2.2 线性范围、检出限和定量限

在优化的测定条件下，以空白红提样品基质提取溶液按照1.3节配制基质匹配标准曲线，按照待测物峰面积为纵坐标（*y*），对应的目标物质量浓度为横坐标（*x*，μg/L）绘制基质匹配标准曲线并计算回归方程。在空白基质中添加痕量标准溶液，以仪器信噪比（*S/N*）为3时所对应的标准溶液质量浓度为仪器检出限，*S/N*为10时所对应的质量浓度为仪器定量限，根据前处理步骤获得各物质的方法检出限（LOD）与方法定量限（LOQ）。结果显示，79种农药在0.1~200 μg/L范围内线性关系良好，相关系数（*r*）均大于0.990；79种农药的LOD和LOQ范围分别为0.01~4.0 μg/kg和0.03~13.0 μg/kg（见附表1）。本方法仅内吸磷、涕灭威、涕灭威亚砜和甲拌磷的LOQ略高于GB 23200.121-2021《食品安全国家标准 植物源性食品中331种农药及其代谢物残留量的测定 液相色谱-质谱联用法》，绝大部分待测物的LOQ明显优于国标要求。

#### 2.2.3 回收率和精密度

以红提、番茄为典型空白样品，以79种农药各个组分1倍定量限、2倍定量限和10倍定量限作为低中高3个水平进行加标回收试验，每个含量水平做6次平行考察精密度。两种基质中79种农药在3个加标水平下的回收率为78.2%~119.8%，相对标准偏差（RSD）均小于15.8%（见表2），表明该方法的准确度和精密度良好，可用于果蔬样品的分析。

**表2:** 79种农药的加标回收率及相对标准偏差（*n*=6）

Compound	Recoveries （RSDs）/%					
	Tomato			Red grape		
	Low	Medium	High	Low	Medium	High
Methamidophos	116.3 （2.6）	96.5 （0.4）	89.7 （4.3）	84.9 （2.9）	81.0 （1.8）	84.3 （4.8）
Methomyl	84.3 （7.9）	98.1 （4.7）	103.9 （4.4）	87.0 （10.5）	102.6 （7.5）	96.6 （9.2）
Metolcarb	99.8 （5.4）	95.7 （5.6）	101.6 （7.7）	81.6 （4.3）	98.8 （4.7）	95.5 （10.6）
Cyromazine	105.0 （0.9）	109.1 （3.6）	108.7 （6.6）	92.7 （1.0）	89.3 （1.4）	92.8 （8.4）
XMC emulsion	97.9 （2.5）	93.5 （3.5）	98.5 （3.7）	84.4 （4.1）	98.4 （4.8）	96.2 （3.0）
Acephate	94.6 （7.3）	104.6 （2.2）	106.4 （6.7）	82.3 （3.5）	85.5 （1.9）	93.8 （11.0）
Carbendazim	83.5 （4.2）	85.8 （5.5）	85.4 （2.1）	89.6 （2.7）	98.5 （0.9）	93.2 （12.9）
Diethyltoluamide	93.1 （1.6）	78.5 （2.9）	78.2 （4.7）	101.3 （3.1）	90.6 （2.0）	82.5 （2.2）
Isoprocarb	95.0 （5.8）	98.7 （2.6）	98.9 （3.7）	88.3 （5.1）	99.5 （2.9）	86.7 （4.5）
Pyrimethanil	105.9 （8.8）	111.1 （3.6）	106.7 （2.9）	83.5 （5.6）	98.0 （2.2）	89.1 （15.8）
Carbaryl	85.8 （5.2）	98.2 （2.9）	93.9 （2.7）	89.3 （2.5）	97.3 （3.0）	92.7 （2.8）
Dinotefuran	116.5 （1.9）	118.6 （2.9）	103.1 （4.9）	80.2 （4.8）	93.4 （2.0）	81.3 （11.0）
Aldicarb sulfoxid	80.5 （2.5）	104.4 （4.7）	102.7 （2.6）	118.2 （5.0）	91.8 （3.2）	86.3 （7.9）
Propoxur	88.2 （7.3）	91.9 （4.6）	83.8 （2.6）	90.2 （6.4）	98.9 （3.9）	94.8 （11.8）
Aldicarb	111.7 （9.1）	115.4 （2.5）	101.6 （5.3）	81.4 （8.5）	95.6 （3.7）	94.4 （4.6）
Omethoate	109.7 （1.4）	110.5 （5.3）	104.5 （3.5）	98.1 （3.7）	90.7 （1.7）	115.2 （2.4）
Atrazine	102.4 （3.1）	104.9 （15.8）	78.7 （4.7）	84.0 （5.5）	101.1 （1.1）	96.7 （5.9）
Carbofuran	100.8 （0.9）	103.7 （2.6）	93.3 （3.5）	86.5 （5.4）	100.2 （1.9）	89.2 （2.7）
Aldicarb-sulfone	93.5 （1.6）	102.0 （1.4）	98.8 （5.8）	81.6 （4.5）	96.1 （2.8）	87.8 （15.1）
Acetamiprid	81.2 （1.6）	116.2 （3.5）	91.0 （3.8）	82.8 （4.1）	99.3 （0.9）	106.9 （3.1）
Monocrotophos	97.8 （6.4）	90.9 （2.5）	92.2 （4.6）	85.3 （5.0）	98.0 （2.2）	94.3 （6.5）
Mevinphos 1	97.7 （7.4）	101.9 （2.8）	96.4 （4.3）	83.8 （5.4）	98.3 （1.8）	96.8 （12.7）
Mevinphos 2	101.6 （7.9）	107.9 （7.3）	94.7 （3.6）	80.2 （4.5）	93.2 （2.2）	91.5 （10.8）
Mercaptodimethur	83.5 （5.8）	82.4 （2.7）	87.9 （6.3）	87.3 （5.5）	97.4 （3.8）	90.1 （14.2）
Picaridin	103.1 （3.6）	118.9 （3.6）	118.6 （11.8）	86.5 （4.6）	98.1 （3.7）	110.9 （7.9）
Dimethoate	89.4 （4.6）	94.1 （5.3）	94.7 （6.3）	115.7 （5.2）	114.3 （15.8）	89.2 （6.3）
3-Hydroxycarbofuran	91.2 （2.6）	97.9 （6.3）	95.0 （5.3）	83.8 （5.6）	94.3 （3.5）	87.4 （5.5）
Pirimicarb	100.1 （2.9）	100.2 （1.3）	88.3 （4.3）	82.0 （4.9）	99.6 （1.3）	110.9 （11.4）
Clomazone	113.3 （3.7）	95.0 （4.3）	88.9 （3.3）	85.1 （5.4）	100.6 （1.4）	102.7 （3.9）
Clothianidin	117.3 （7.5）	98.7 （2.7）	98.3 （1.8）	89.9 （5.1）	99.6 （1.9）	81.3 （12.9）
Thiacloprid	106.7 （4.2）	85.4 （2.6）	78.8 （4.5）	86.2 （4.6）	100.8 （1.3）	82.5 （14.3）
Metasystox	107.7 （3.9）	100.4 （2.1）	94.8 （3.6）	82.6 （5.5）	87.0 （2.4）	89.7 （11.2）
Imidacloprid	114.7 （6.6）	116.2 （7.8）	115.6 （10.6）	83.5 （4.7）	97.4 （2.2）	109.0 （11.5）
Phorate	79.8 （2.8）	113.3 （4.4）	118.9 （4.1）	89.5 （13.9）	94.0 （7.7）	84.7 （4.8）
Imidaclothiz	112.9 （3.6）	94.5 （5.5）	117.6 （3.7）	101.3 （3.8）	92.5 （4.7）	92.6 （9.2）
Demeton-*S*-methyl sulfone	98.5 （4.4）	96.4 （4.3）	99.5 （6.5）	82.6 （6.3）	99.7 （1.3）	110.9 （10.6）
Nitenpyram	118.6 （4.7）	84.7 （3.8）	98.4 （6.5）	102.4 （12.2）	80.4 （4.2）	106.0 （8.4）
Cadusafos	101.3 （2.8）	78.9 （2.8）	94.7 （2.9）	86.5 （7.9）	93.8 （3.7）	108.0 （3.0）
Phorate sulfoxide	94.0 （1.8）	98.7 （4.3）	93.9 （2.9）	90.0 （4.2）	102.8 （1.8）	96.4 （11.0）
Sulfoxaflor	86.8 （5.6）	98.6 （6.4）	104.1 （7.8）	81.6 （9.0）	102.0 （6.2）	84.3 （12.9）
Metalaxyl	98.7 （2.3）	97.2 （2.9）	81.9 （4.5）	86.6 （4.7）	100.9 （1.4）	96.6 （2.2）
Dodemorph	93.5 （2.9）	78.2 （2.9）	104.1 （2.9）	83.7 （5.9）	99.4 （0.9）	95.5 （4.5）
Pendimethalin	95.1 （2.9）	119.8 （2.9）	118.3 （2.9）	114.0 （11.2）	95.7 （4.8）	104.2 （14.8）
Vamidothion	89.7 （2.9）	85.4 （2.9）	79.9 （1.9）	81.1 （4.3）	98.5 （1.0）	96.2 （2.8）
Flupyradifurone	107.5 （5.8）	95.1 （5.3）	116.4 （6.4）	83.5 （5.9）	98.2 （2.7）	93.8 （11.0）
Myclobutanil	108.7 （1.8）	107.9 （4.3）	109.1 （3.2）	86.0 （6.0）	102.5 （2.1）	93.2 （7.9）
Thiamethoxam	115.6 （4.5）	101.6 （2.4）	100.2 （5.3）	80.6 （4.9）	91.7 （3.1）	82.5 （11.8）
Phorate sulfone	91.9 （2.6）	99.5 （3.9）	97.3 （4.3）	87.9 （7.8）	100.9 （3.1）	86.7 （4.6）
Triadimefon	109.9 （3.8）	111.6 （2.9）	114.3 （2.8）	82.8 （6.4）	100.5 （3.3）	89.1 （2.4）
Mefenacet	96.8 （3.3）	92.1 （4.3）	91.5 （4.7）	80.8 （5.3）	94.6 （3.6）	92.7 （5.9）
Bifenazate	97.8 （1.5）	110.7 （3.2）	101.4 （2.3）	82.4 （4.9）	92.9 （3.8）	104.5 （2.7）
Clofentezine	118.1 （3.7）	113.8 （4.2）	113.6 （4.3）	78.3 （5.6）	92.6 （4.9）	100.9 （15.1）
Fenamiphos	92.4 （4.3）	79.4 （1.8）	90.1 （2.3）	85.3 （3.6）	98.5 （1.1）	94.8 （3.1）
Tebuconazole	110.4 （4.1）	104.0 （2.1）	115.5 （3.4）	84.2 （6.4）	100.3 （2.5）	94.4 （6.5）
Azinphos-methyl	97.6 （7.4）	116.9 （6.6）	113.5 （4.3）	78.2 （15.8）	84.8 （3.0）	108.6 （12.7）
Fenamiphos sulfoxide	105.8 （4.2）	108.5 （4.6）	96.0 （7.4）	86.0 （4.3）	101.1 （1.1）	96.7 （10.8）
Sulfadiazine	89.4 （9.6）	119.8 （8.3）	102.1 （7.5）	96.9 （14.0）	113.1 （10.3）	112.4 （14.2）
Benalaxyl	99.3 （4.3）	99.5 （3.2）	96.8 （6.4）	85.4 （5.2）	99.2 （2.7）	87.8 （7.9）
Pyrethrin	94.0 （4.3）	95.7 （3.7）	98.8 （2.6）	104.7 （12.0）	79.3 （6.9）	109.3 （6.3）
Pirimiphos ethyl	92.9 （2.4）	80.7 （1.1）	109.5 （4.2）	89.6 （2.9）	98.7 （1.2）	94.3 （5.5）
Fenamiphos sulfone	110.8 （3.1）	111.8 （0.7）	80.4 （7.3）	84.8 （4.9）	101.1 （1.1）	96.8 （11.4）
Propiconazole	104.4 （3.6）	112.8 （4.6）	113.2 （3.8）	81.1 （3.7）	99.1 （2.5）	91.5 （3.9）
Thiophanate-methyl	114.9 （3.8）	115.2 （1.6）	78.2 （6.4）	117.3 （4.7）	92.9 （1.1）	90.1 （12.9）
Oxazone	92.2 （7.7）	108.8 （7.4）	105.4 （9.3）	112.6 （5.2）	93.0 （4.0）	115.9 （14.3）
Etoxazole	92.0 （2.1）	97.4 （1.5）	113.4 （6.3）	82.4 （2.6）	93.6 （0.9）	89.2 （11.2）
Sanmite	97.8 （1.5）	84.7 （6.4）	84.8 （10.7）	81.3 （8.3）	93.8 （3.5）	87.4 （19.5）
Paichongding 1	114.1 （5.5）	111.7 （3.7）	110.1 （4.6）	92.5 （5.3）	84.4 （2.1）	114.0 （4.8）
Paichongding 2	98.2 （9.4）	111.8 （6.2）	108.9 （8.4）	101.2 （11.2）	91.6 （7.9）	100.0 （9.2）
Propargite	80.3 （4.4）	79.4 （4.3）	78.5 （6.8）	79.8 （7.1）	90.8 （5.7）	100.8 （10.6）
Prochloraz	91.3 （4.2）	114.3 （3.1）	97.4 （6.6）	78.9 （6.2）	86.5 （3.7）	82.5 （8.4）
Pyraclostrobin	97.4 （4.6）	107.7 （2.9）	108.8 （3.1）	83.9 （5.3）	97.2 （2.3）	89.7 （3.0）
Dimethomorph 2	108.4 （3.1）	80.7 （4.7）	79.8 （4.3）	105.0 （7.0）	114.0 （7.2）	119.2 （11.0）
Dimethomorph 1	107.8 （5.1）	81.2 （4.9）	81.6 （7.2）	80.9 （4.9）	98.7 （2.8）	84.7 （12.9）
Difenoconazole	104.4 （2.1）	111.4 （1.8）	95.2 （3.8）	80.5 （5.6）	99.5 （1.6）	92.6 （2.2）
Spirodiclofen	90.1 （2.8）	79.8 （4.0）	85.5 （5.7）	78.2 （9.4）	82.1 （4.5）	96.7 （4.5）
Cyflumetofen	96.1 （4.6）	78.5 （3.2）	79.3 （6.8）	109.5 （6.2）	89.0 （3.4）	102.3 （15.8）
Abamectin	88.7 （1.5）	118.5 （3.3）	117.7 （2.2）	78.6 （3.9）	87.7 （3.8）	78.2 （2.7）
Fluazinam	90.8 （8.8）	113.1 （9.4）	90.4 （5.4）	78.5 （10.2）	89.7 （3.1）	78.6 （11.0）
Demeton	79.8 （8.0）	95.8 （2.7）	105.9 （3.3）	84.4 （5.8）	104.9 （9.5）	79.0 （6.6）

### 2.3 实际样品测定

用建立的方法对采自湖南省10个地区的80份市售果蔬样本进行检测。在80份果蔬样本中，有31份样本检出19种农药残留，其中13份样本中检出噻虫胺，含量范围为0.017~1.92 mg/kg；11份样本中检出啶虫脒，含量范围为0.012~0.67 mg/kg；7份样本中检出噻虫嗪，含量范围为0.021~2.62 mg/kg；3种农药检出率分别为16.3%、13.8%、8.8%。典型样品的总离子流图见图3。

**图3:**
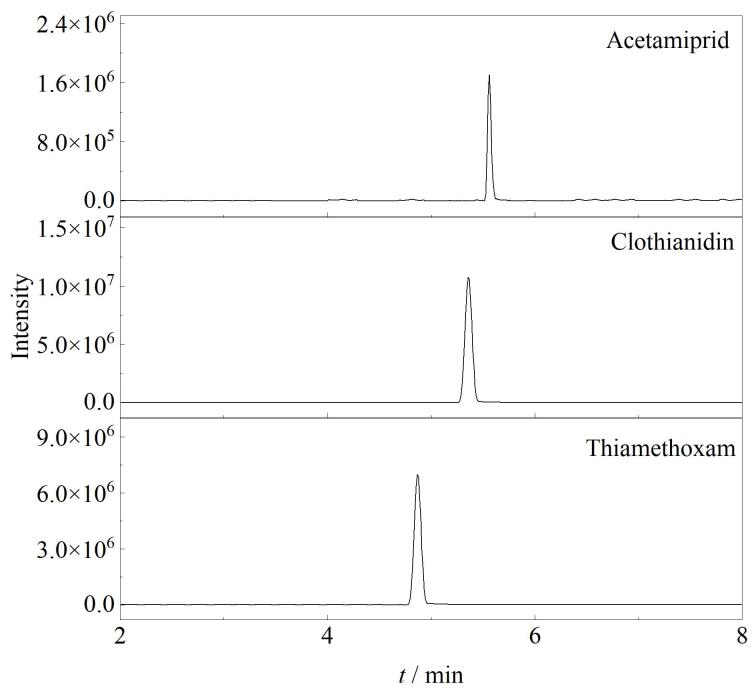
典型样品的总离子流图

噻虫胺和噻虫嗪均属于新型烟碱类杀虫剂（neonicotinoid insecticides，NEOs），这类农药具有活性高、安全性好、广谱性和见效快等特点，已成为替代高毒性高生物蓄积的有机磷和有机氯类杀虫剂的主要品种^［21］^，这也是NEOs在果蔬中检出率较高的原因。有研究表明，NEOs暴露干扰机体正常激素平衡，影响生殖和新陈代谢等^［22］^，尤其与早产和新生儿畸形等不良妊娠结果之间存在显著关联^［23］^，有必要对NEOs进行慢性膳食摄入风险值（%ADI）评估。慢性健康风险与%ADI呈正相关，即%ADI越高，健康风险越大，当%ADI≤100%时，健康风险处于可接受水平；而当%ADI＞100%时，表明不可接受。%ADI计算公式^［24］^如下：


$$\%ADI=\frac{Rm\times \;F}{bw\times ADI}\times 100\%$$ （1）

式中Rm为样本中农药残留平均检测数值，本文用最大农药残留量代替；*F*为食物消费量，根据《2024湖南调查年鉴》查得湖南省2023年人均蔬菜和水果的消费量分别为0.317 kg/d、0.159 kg/d；bw为一般人群的体重53.23 kg^［25］^；以及查得噻虫胺和噻虫嗪每日允许摄入量（ADI）分别为0.10 mg/kg bw和0.08 mg/kg bw，计算获得噻虫胺在水果和蔬菜中的%ADI为5.74%和0.36%，噻虫嗪在水果和蔬菜中的%ADI分别为0.40%和19.50%，二者在蔬菜和水果中的%ADI均低于100%，风险较低。因此测得的阳性样本含量风险可接受。

此外，检出农药残留种类较多的样本是酸豇豆和豇豆，分别检出了8种和7种农药残留。豇豆在生长过程中易受到隐藏于豆荚内的害虫侵害，因此会用到多种新型农药，酸豆角是豇豆的腌制加工品，说明腌制工艺对豇豆中残留农药的降解作用有限。另一方面，79种农药中有13种农药尚未纳入GB 2763-2021《食品安全国家标准 食品中农药最大残留限量》范围内，因此补充食物中农药残留限量标准十分有必要。

## 3 结论

本研究建立了果蔬中79种农药残留同时测定的方法，样品采用QuEChERS法净化，UPLC-MS/MS测定，简便快捷，方法灵敏度高，回收率和精密度良好。以该方法对80份果蔬样品开展检测，烟碱类农药残留检出率高，初步风险评估显示湖南省果蔬中的烟碱类农药残留在可允许接受的范围内，膳食风险较低，但仍需加强对植物源性腌制食品农药残留的监管力度，并持续完善食品中农药残留国家标准的监测体系，重点应扩大对新型农药的监测范围。
